# Optimal Delay Time of CT Perfusion for Predicting Cerebral Parenchymal Hematoma After Intra-Arterial tPA Treatment

**DOI:** 10.3389/fneur.2018.00680

**Published:** 2018-08-21

**Authors:** Bing Wu, Nan Liu, Max Wintermark, Mark W. Parsons, Hui Chen, Longting Lin, Shuai Zhou, Gang Hu, Yongwei Zhang, Jun Hu, Ying Li, Zihua Su, Xinhuai Wu, Guangming Zhu

**Affiliations:** ^1^Department of Radiology, PLA Army General Hospital, Beijing, China; ^2^Department of Neurology, PLA Army General Hospital, Beijing, China; ^3^Neuroradiology Section, Department of Radiology, Stanford University, Stanford, CA, United States; ^4^School of Medicine and Public Health, University of Newcastle, Newcastle, NSW, Australia; ^5^Department of Neurology, John Hunter Hospital, University of Newcastle, Newcastle, NSW, Australia; ^6^Inner Mongolia Medical University Hohhot, China; ^7^Department of Neurology, Changhai Hospital, Second Military Medical University, Shanghai, China; ^8^Department of Neurology, Southwest Hospital, Third Military Medical University, Chongqing, China; ^9^GE Healthcare, Beijing, China

**Keywords:** stroke, hemorrhage transformation, CT scan, perfusion imaging, delay time

## Abstract

**Background and Purpose:** Cerebral hemorrhage is a serious potential complication of stroke revascularization, especially in patients receiving intra-arterial tissue-type plasminogen activator (tPA) therapy. We investigated the optimal pre-intervention delay time (DT) of computed tomography perfusion (CTP) measurement to predict cerebral parenchymal hematoma (PH) in acute ischemic stroke (AIS) patients after intra-arterial tissue plasminogen activator (tPA) treatment.

**Methods:** The study population consisted of a series of patients with AIS who received intra-arterial tPA treatment and had CTP and follow-up computed tomography/magnetic resonance imaging (CT/MRI) to identify hemorrhagic transformation. The association of increasing DT thresholds (>2, >4, >6, >8, and >10 s) with PH was examined using receiver operating characteristic (ROC) analysis and logistic regression.

**Results:** Of 94 patients, 23 developed PH on follow-up imaging. Receiver operating characteristic analysis revealed that the greatest area under the curve for predicting PH occurred at DT > 4 s (area under the curve, 0.66). At this threshold of > 4 s, DT lesion volume ≥ 30.85 mL optimally predicted PH with 70% sensitivity and 59% specificity. DT > 4 s volume was independently predictive of PH in a multivariate logistic regression model (*P* < 0.05).

**Conclusions:** DT > 4 s was the parameter most strongly associated with PH. The volume of moderate, not severe, hypo-perfusion on DT is more strongly associated and may allow better prediction of PH after intra-arterial tPA thrombolysis.

## Introduction

While significant advances have been made regarding emergent treatment of acute ischemic stroke (AIS), the different therapeutic revascularization options remain associated with an increased risk of hemorrhage. While intravenous tissue-type plasminogen activator (tPA) is effective at reanalyzing more distal thrombi (1, 2), endovascular reperfusion therapy in a 6 or 12 h time window, can be effective for those patients with more proximal intracranial artery occlusion (3–6). The revascularization increases the risk of Hemorrhagic transformation (HT) (7–9), and HT is nearly 5 times more common for patients receiving intravenous thrombolysis compared to controls, and even more for patients receiving intra-arterial thrombolysis (10). Symptomatic intracranial hemorrhage (sICH) transformation or cerebral parenchymal hematoma (PH) is the most serious complication after revascularization therapy. The identification and possible exclusion of patients at high risk for sICH or PH will significantly reduce the complication rate (11).

Several clinical risk factors, such as age, diabetes mellitus, infarct volume, and anticoagulant or antiplatelet therapy are associated with HT. Computed tomography (CT) and magnetic resonance imaging (MRI), including perfusion imaging, provide detailed assessment of acute stroke pathophysiology, and has led to the establishment of imaging predictive parameters for HT after thrombolysis. Relative cerebral blood flow (rCBF), relative mean transit time, cerebral blood volume (CBV), Tmax, delay time (DT), and permeability parameters have been found to be associated with hemorrhagic transformation (12–16). A prior study by Yassi et al (15) showed that extremely long Tmax was independently predictive of PH for the patient with or without thrombolysis. However, these studies focused on patients who received intravenous tPA, rather than on patients who received intra-arterial tPA treatment.

In this study, we sought to identify the optimal pre-intervention DT parameter for prediction of PH after AIS intra-arterial tPA therapy.

## Methods

### Patients

**Figure 1:**
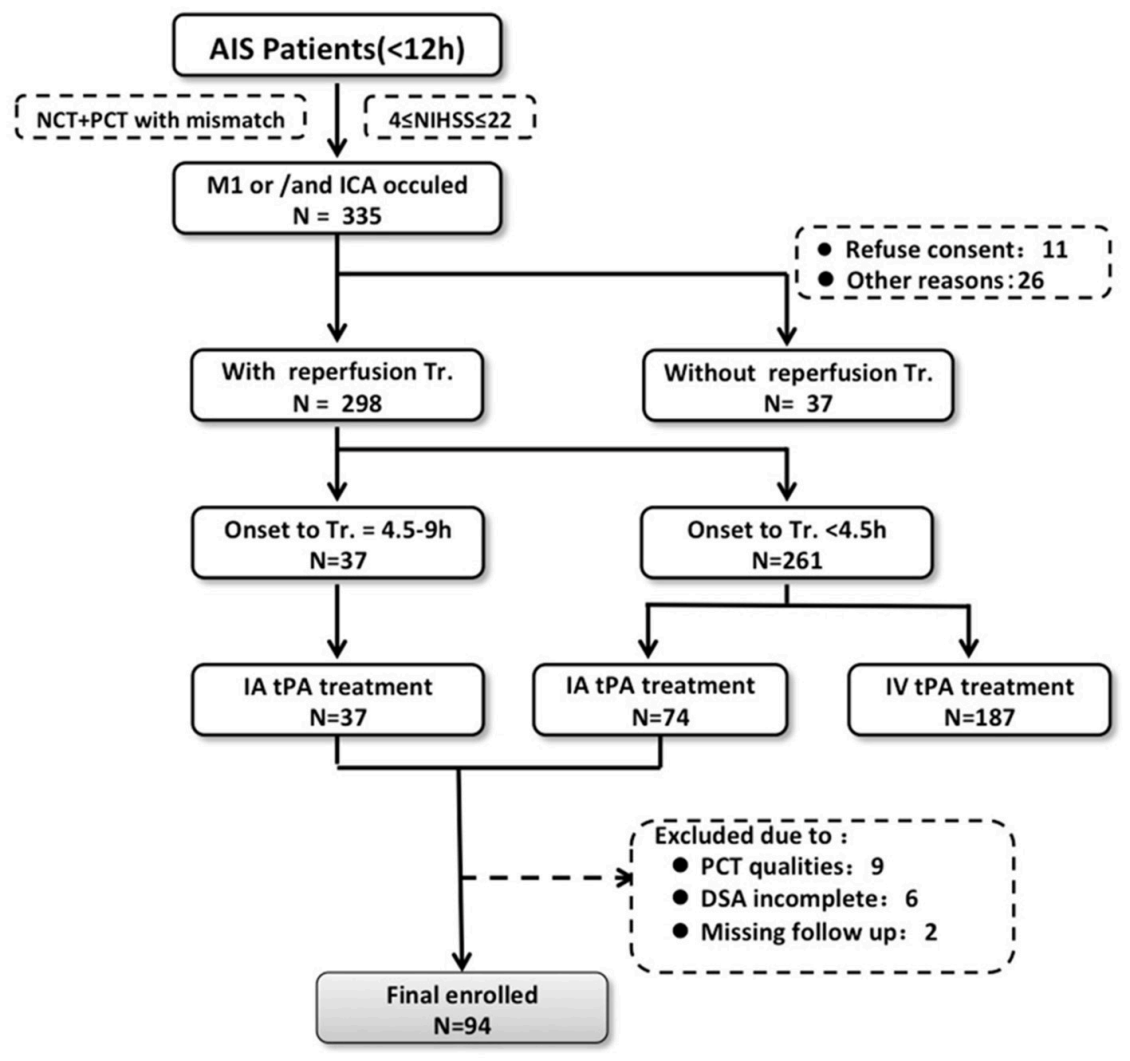
Flow chart outlining patient selection and exclusion criteria. AIS indicates acute ischemic stroke; DSA, digital subtraction angiography; IA, intra-arterial; IV tPA, intravenous tissue-type plasminogen activator; NCT, noncontrast-CT; NIHSS, National Institutes of Health Stroke Scale; CTP, computed tomography perfusion; and Tr, treatment.

In this study, the clinical and imaging data were obtained from 3 participating institutions: the PLA Army General Hospital, Beijing; Changhai Hospital, Shanghai; and Southwest Hospital, Chongqing. All data contributed to the study were completely anonymized. The institutional review boards of the three institutions approved the study. Consecutive patients with signs and symptoms suggesting hemispheric stroke from January 2011 to January 2014 were retrospectively identified. Inclusion criteria were as follows: (1) AIS with occlusion of the M1 segment of the middle cerebral artery, the internal carotid, or both; (2) an admission National Institutes of Health Stroke Scale (NIHSS) score between 4 and 22; (3) with CT imaging indicating stroke, including non-contrast-CT, CT angilgraphy (CTA), and CT perfusion (CTP), upon admission; (4) intra-arterial tPA thrombolysis with <12 h from onset; (5) availability of MRIs or CT scans taken within 7 days after therapy to assess HT. The patients who received IV-tPA were not included in the study, considering data consistency. A flow chart delineating patient selection is shown in Figure 1. The demographic and clinical variables were recorded as follows: age, sex, medical history, vascular risk factors, routine blood tests, time from onset to imaging, time from symptom onset to treatment, NIHSS score upon admission, and modified Rankin Score (mRS) at 90 days. The mRS was assessed in the outpatient clinic or by the telephone and the death was coded as 6. Stroke mechanisms were subtyped using the TOAST (Trial of Org 10172 in Acute Stroke Treatment) classification and were diagnosed by 2 stroke neurologists (N.L. and H.C.) in consensus.

### Imaging protocol

CTP studies were all obtained on 64-slice CT scanners. Each CTP study involved successive gantry rotations performed in cine mode with 45 time-points acquired each 1.33 s (total acquisition, 60 s), with intravenous administration of 40–50 mL of iodinated contrast material (Ultravist 370; Bayer HealthCare; Berlin, Germany) at an injection rate of 4–5 mL/s followed by a 40-mL saline push. Total CTP coverage was 40 mm. Acquisition parameters were 80 kVp and 100 mAs.

Digital subtraction angiography (DSA) was performed using a biplane cerebral angiographic system. Images were acquired during injection of the internal and external carotid arteries and ≥1 vertebral artery. Imaging was performed through the entire arterial and venous phases to evaluate the collateral circulation. All patients underwent intra-arterial tPA thrombolysis without mechanical embolectomy at the discretion of the attending neurologist (Y.Z, J.H., and G.Z.).

### Imaging processing

All perfusion CT data were analyzed with the commercial software (MiStar, Apollo Medical Imaging Technology) (17, 18). Perfusion data were processed using a single value deconvolution algorithm with delay and dispersion correction. The actual delay time (DT) was calculated by a modified singular value deconvolution approach by looping through a series of DT values (19). The cerebral blood flow and cerebral blood volume were determined by the peak height and area under the curve of the input residue function. Arterial input function and venous outflow function were automatically selected by the software from the non-stroke middle cerebral artery/anterior cerebral artery and superior sagittal sinus, respectively. The volume of increasing DT thresholds (2, >4, >6, >8, and >10 s), the relative cerebral blood flow < 40% within the DT >3 s, and cerebral blood volume < 2 mL/100 g within the DT >3 s, were automatically calculated (17).

Receiver operating characteristic (ROC) analysis was performed using PH as the outcome variable, and the lesion volume was defined using a particular DT, CBV, or rCBF threshold in each individual patient. These thresholds were iterated across the range of values present in the data to determine the threshold for each parameter that generated the highest area under the curve (AUC). This optimal threshold was taken forward in the analysis to compare the sensitivity and specificity of each perfusion parameter. The Youden Index (sensitivity+specificity−1) was then calculated for this optimized threshold to determine the optimal volume of rCBF, CBV, and DT to predict the development of PH.

It has previously been demonstrated that poor baseline collaterals and successful therapeutic recanalization may result in clinically significant hemorrhagic complications (20). The angiographic collateral was scaled as follows: grade 0 (no collaterals visible to the ischemic site), 1 (slow collaterals to the periphery of the ischemic site with persistence of some of the defects), 2 (rapid collaterals to the periphery of ischemic site with persistence of some of the defects and to only a portion of the ischemic territory), 3 (collaterals with slow but complete angiographic blood flow of the ischemic bed by the late venous phase), and 4 (complete and rapid collateral blood flow to the vascular bed in the entire ischemic territory by retrograde perfusion). Vascular reperfusion was graded based on the Thrombolysis in Cerebral Infarction (TICI) classification: 0 (no perfusion), 1 (penetration with minimal perfusion), 2a (less than 67% perfusion); 2b (more than 67% perfusion), and 3 (complete perfusion of the affected vascular territory). Reperfusion status was classified as ER+ (positive for early reperfusion; TICI score 2b to 3 within 12 h of symptom onset) or ER–. The angiographic collateral and TICI scores were rated by consensus of a neurologist (G.Z.) and a neuroradiologist (B.W.). The influence of collaterals and recanalization on PH was analyzed in distinct case scenarios relative to baseline collateral grade at angiography (0–1 vs. 2–4) and recanalization (TICI scale, ER+ vs. ER–): (1) good collaterals and no recanalization, (2) poor collaterals and no recanalization, (3) good collaterals and successful recanalization, and (4) poor collaterals with successful recanalization.

### Outcome measurement

All patients received follow-up CT or MRI as part of the routine. However the time interval depended on the patient's condition. Follow-up imaging (MRI or CT within 7 days) was independently assessed for hemorrhagic transformation by 2 stroke neurologists (N.L. and H.C.), who then reached consensus using the European Cooperative Acute Stroke Study (ECASS) scoring system (21). This classifies hemorrhagic infarction 1 (HI1) as small petechiae along the periphery of the infarct region, hemorrhagic infarction 2 (HI2) as confluent petechiae within the infarct, without space-occupying effect, parenchymal hemorrhage 1 (PH1) as bleeding ≤30% of the infarcted area, with mild space-occupying effect, and parenchymal hemorrhage 2 (PH2) as bleeding >30% of the infarcted area, with space-occupying effect. PH included both PH1 and PH2. The readers of neuroradiological imaging were blinded to all clinical data.

### Statistical analyses

Statistical analysis was performed using commercially available IBM SPSS Statistics (version 22.0.0.0; IBM Corp, Armonk, NY). The Mann–Whitney U–test was used to determine the association between baseline clinical characteristics or imaging parameters with parenchymal hemorrhage outcome. ROC analysis was performed to determine the optimal CTP parameter for prediction of PH. Sensitivity, specificity, positive predictive value, negative predictive value, and likelihood ratios were determined for each parameter at different volumetric thresholds. The best performing CTP parameters in ROC analysis were subsequently tested in a multivariate logistic regression model including age, baseline NIHSS score, and poor collaterals and recanalization. The other variables were tested in a univariate model logistic regression. The variable would be added to the multivariate model when it was statistically significant in univariate model. For perfusion lesions, we divided the lesion volumes into 4 groups according to interquartile cutoff points of the distribution of volumes of perfusion delay. Correlation between DT >2 s, >4 s, >6 s, >8 s, and >10 s were also analyzed with the correlation coefficient matrix. *P* < 0.05 was considered statistically significant.

## Results

Of 111 consecutive ischemic stroke patients imaged with multi-modal CT and intra-arterial thrombolysis treatment, 94 were included in the analysis. The reasons for exclusion were severely motion-degraded perfusion data (*n* = 9), incomplete DSA imaging (*n* = 6), and 2 patients could not be contacted during follow-up. Follow-up MRI and CT were performed in 75 and 19 patients, respectively. PH occurred in 23 patients overall (24.5%). Table 1 shows baseline clinical characteristics, stroke risk factors, and time to imaging for the study patients.

**Table 1:** Demographic parameters and other relevant information (*n* = 94).

**Clinical and imaging characteristics**	**No PH**	**PH**	***P-*value**
	***N* = 71**	***N* = 23**	
Male,%	40 (56.3)	14 (60.9)	0.8101
Age, years	68.0 (53.0–77.0)	68.0 (55.0–73.0)	0.8156
NIHSS score at admission	16.0 (11.0–19.0)	17.0 (13.0–19.0)	0.2254
Hypertension,%	44 (62.0)	14 (60.9)	0.9247
Diabetes mellitus,%	10 (14.1)	4 (17.4)	0.7398
Hyperlipidemia,%	24 (33.8)	3 (13.0)	0.0666
Atrial fibrillation,%	19 (27.1)	3 (13.0)	0.2581
CAD,%	15 (22.1)	3 (13.0)	0.5456
Current statin administration,%	19 (26.8)	3 (13.0)	0.2587
Stroke mechanism			
Cardioembolic stroke,%	23 (32.4)	5 (21.7)	0.1644
Large artery disease,%	20 (28.2)	12 (52.2)	
Other type of stroke,%	10 (14.1)	1 (4.4)	
Undetermined Categories,%	18 (25.4)	5 (21.7)	
Time from onset to CT imaging, hours	5.5 (3.5–7.8)	6.0 (4.8–11.5)	0.3011
ASPECTS score on NCT	8.0 (7.0–9.0)	7.0 (6.0–9.0)	0.1003
HMCAS on NCT, %	35 (55.6)	17 (73.9)	0.1425
Collateral Flow Scores	2.0 (1.0–3.0)	2.0 (2.0–3.0)	0.3106
DT>2 s, mL	55.7 (47.0–70.7)	59.8 (46.2–70.2)	0.4442
DT>4 s, mL	27.7 (17.0–40.0)	36.6 (25.3–51.7)	0.0243^*^
DT>6 s, mL	11.1 (2.2–21.3)	17.6 (8.1–34.2)	0.054
DT>8 s, mL	5.1 (0.3–10.1)	6.7 (2.5–19.9)	0.0854
DT>10 s, mL	1.6 (0.0–4.6)	2.2 (0.3–10.6)	0.1157
rCBF < 40%, mL	16.2 (4.3–23.4)	16.9 (6.1–30.5)	0.2132
CBV < 2, mL	17.2 (3.5–32.3)	21.9 (5.1–32.5)	0.7447
Site of occlusion			
ICA, %	5 (7.0)	3 (13.0)	0.2687
M1, %	53 (74.7)	13 (56.5)	
ICA &M1, %	13 (18.3)	7 (30.4)	
Successful recanalization (TIMI:2-3)	57 (80.3)	19 (82.6)	0.8053

*ASPECTS indicates Alberta Stroke Program Early CT Score; CAD, coronary artery disease; CT, computed tomography; HMCAS, hyperdense middle cerebral artery sign; NCT, noncontrast-CT; NIHSS, National Institutes of Health Stroke Scale; DT, delay time; PH, parenchymal hematoma; rCBF, relative cerebral bold flow, CBV, cerebral blood volume; ICA, internal carotid artery; M1, middle cerebral artery; TIMI, thrombolysis in myocardial infarct*.

* *P < 0.05*.

Male percent, atrial fibrillation, hyperlipidemia, ASPECTS on NCT, collateral flow score, and volume of CBV <2 mL/100 g were significantly different among types of HT, but not associated with PH, as shown in Table 1. The volume of DT>4 s was significantly associated with PH.

The ROC analysis for association of PH across the range of values in each CTP parameter identified DT>4 s as the optimal threshold for further analysis (area under the curve = 0.657; *P* = 0.024), followed by rCBF <40% (area under the curve = 0.587; *P* = 0.212), and CBV < 2 mL/100 g (area under the curve = 0.523; *P* = 0.742). Mean DT>4 s volume was 27.7 mL (interquartile range [IQR], 17.0–40.0) in the no-PH group and 36.6 mL (IQR, 25.3–51.7) in the PH group (*P* = 0.024; Mann–Whitney U test) (Table 2).

**Table 2:** Receiver operating characteristic analysis.

	**PH**			
	**AUC**	**95% CI**		***P*-value**
DT>2 s	0.554	0.415	0.692	0.442
DT>4 s	0.657	0.525	0.789	0.024^*^
DT>6 s	0.634	0.498	0.77	0.054
DT>8 s	0.620	0.484	0.755	0.086
DT>10 s	0.608	0.47	0.747	0.120
rCBF <40%	0.587	0.446	0.728	0.212
CBV <2	0.523	0.392	0.654	0.742

DT indicates delay time; AUC, area under the curve; CI, confidence interval; rCBF, relative cerebral bold flow, CBV, cerebral blood volume;

* *P < 0.05*.

Based on the ROC analysis and Youden Index, the optimal volume of DT>4 s for the association with PH was ≥30.4 mL. There were 49 of 94 (52%) patients with DT>4 s volumes of <30.4 mL, of whom 7 (14.3%) patients developed PH compared with the overall rate of 24.5%. This indicated a low risk of PH in this group, with a negative predictive value of 0.86 (95% confidence interval, 0.73–0.94) and a negative likelihood ratio of 0.51(0.27–0.98). For DT >4 s volumes ≥30.4 mL, the sensitivity for PH was 0.70 (0.49–0.84), the specificity was 0.59 (0.48–0.70), and the positive likelihood ratio was 1.70(1.15–2.51).

A large area of moderate, not severe, perfusion delay (DT >4 s) on the pretreatment CTP were independently associated with PH; compared with patients with the lowest DT >4 s volume quartile, those with second, third, and fourth quartile were approximately 1.3, 3.3, and 3.6 times more likely to develop PH, respectively.

We also found significant correlations between DT >2 s, >4 s, >6 s, >8 s, and >10 s, with correlation coefficients ranging from 0.906 to 0.992.

In backward stepwise elimination logistic regression, including age, baseline NIHSS score, poor collaterals and recanalization, and DT >4 s, only the volume of DT >4 s was independently associated with PH (odds ratio (OR) 1.04 per 1 mL increase in DT >4 s volume [95% confidence interval (CI), 1.01–1.06]; *P* = 0.011). Table 3 shows the results of the logistic regression models and the ORs for PH.

**Table 3:** Logistic regression analysis for PH.

**Variable**	**Multivariate Model**	
	**Odds ratio (CI)**	***P*-value**
NIHSS	1.09 (0.98–1.21)	0.110
Age	0.99 (0.95–1.03)	0.597
DT >4 s, mL^a^	1.04 (1.01–1.06)	0.011^*^
Poor collaterals and recanalization	1.04 (0.59–1.82)	0.901
CTP^**^		
DT > 4 s		
Q2	1.33 (0.26–6.74)	0.728
Q3	3.33 (0.76–14.66)	0.111
Q4	3.56 (0.80–15.72)	0.094
DT > 6 s		
Q2	3.50 (0.63–19.54)	0.153
Q3	4.32 (0.79–23.59)	0.091
Q4	5.60 (1.04–30.20)	0.045^*^
DT > 8 s		
Q2	2.75 (0.61–12.29)	0.187
Q3	1.75 (0.37–8.37)	0.481
Q4	3.56 (0.80–15.72)	0.094
DT > 10 s		
Q2	3.50 (0.63–19.54)	0.153
Q3	4.32 (0.79–23.59)	0.091
Q4	5.60 (1.04–30.20)	0.045^*^

*CI indicates 95% confidence interval; CTP, CT perfusion imaging; DT, delay time; and NIHSS, National Institutes of Health Stroke Scale*.

a *Odds ratio (OR) given for each 1 mL increase in DT >4 s volume*.

* *P < 0.05*.

** *Compared with Q1*.

**Figure 2:**
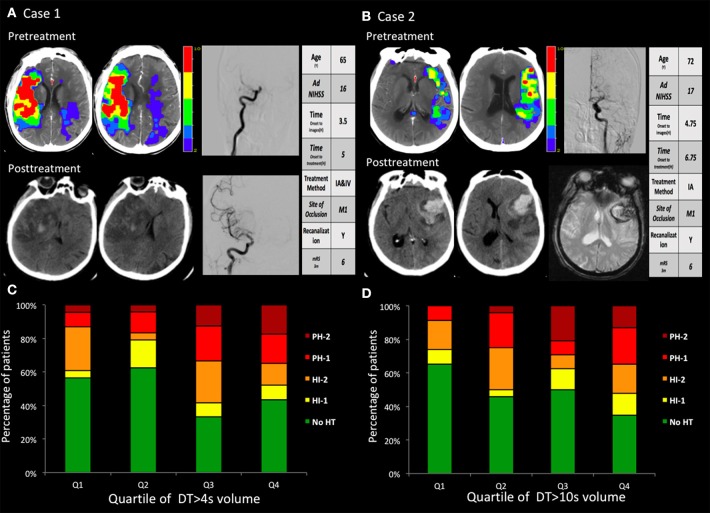
**(A,B)** two cases with hemorrhagic transformation. **(A)** Pretreatment CTP images show a marked large area with perfusion delay. The day after recanalization with intravenous and endovascular treatment, CT findings revealed a PH. The PH was located within the mildly and severely hypoperfused regions. **(B)** Pretreatment CTP images show small areas with large mild perfusion delay and small severe perfusion delay. After recanalization with endovascular treatment, CT and GRE findings revealed a large area of PH. The PH was located within the mildly hypoperfused regions. **(C,D)** The volume in the regions with baseline mild and severe perfusion delay (DT >4 s and DT >10 s) correlated with the PH. Q1 represents the patients with the lowest quartile volume, whereas Q4 indicates patients with the highest quartile volume.

Two illustrative cases with areas of DTs, with subsequent PH after intra-arterial thrombolysis corresponding to the site of abnormal DT are shown in Figure 2.

Based on ECASS hemorrhagic transformation classification, the Supplementary Table 1 shows baseline clinical characteristics, stroke risk factors, and time to imaging for the study patients. The died cases (rate) post-procedure of No HT, HI1, HI2, PH1, and PH2 were 12(26%), 3(33%), 3(19%), 2(14%), and 4(44%), respectively. The ROC analysis for association of any HT across the range of values in each CTP parameter identified rCBF <40% (area under the curve = 0.632; *P* = 0.027), and CBV <2 mL /100 g (area under the curve = 0.650; *P* = 0.012). It has previously been demonstrated that rCBF <40% or CBV <2 mL /100 g on CTP corresponds closely to ischemic core (as shown in Supplementary Table 2). Although rCBF <40% or CBV < 2 mL /100 g could not be significantly associated with the PH in ROC analysis, they were significantly associated with any HT.

## Discussion

Our study has three main findings. Firstly, moderate hypo-perfusion (DT >4 s) predicted PH in patients with endovascular thrombolysis better than other DT values. Secondly, DT >4 s was better than rCBF < 40% or CBV < 2 mL /100 g for PH prediction after IA tPA thrombolysis. Thirdly, the volume of DT>4 s was an independent factor to predict PH, which was more significant than collateral grading and recanalization status.

Although clinical factors are useful in the decision making process before IA tPA thrombolysis administration, in practice, most of these are insufficient in isolation to predict PH. The non-contrast CT before treatment is useful to exclude patients with more than 1/3 of the MCA territory infarct, but of limited value to detect patients at risk of PH in the remaining patients who go on to receive treatment. Although MRI is not routinely available in the acute setting, MRI-based parameters for prediction of post-thrombolysis HT were found in many previous studies, including diffusion-weighted imaging–based lesion volume, severe hypoperfusion measured by high Tmax (22), regional very low CBV (23), and increased permeability (24–26).

CTP is widely available and rapidly accessible in most stroke centers, and thus lends itself to the clinical decision-making process. In this study, the reason we chose DT, instead of Tmax, was that the Tmax value could be dependent on various factors including arterial delay and dispersion and tissue transit time and dispersion. To compensate for arterial delay and dispersion effects, a vascular transport model involving an arterial transport function with a delay time and a relative dispersion has been proposed (19). Thus, there is marked variability in lesion volume prediction among various deconvolution techniques (19, 27).

This study demonstrated that moderate hypo-perfusion (DT >4 s) predicted PH in patients with endovascular thrombolysis better than other DT values. A prior study by Yassi et al. (15), correlating Tmax and PH, showed that extreme hypo-perfusion lesion (Tmax >14 s volumes of >5 mL) and thrombolysis were both independently predictive of PH for the patient with or without thrombolysis. OR values of the Tmax >14 s volumes and the thrombolysis were 4.3 and 10.1, respectively. Thus, thrombolysis had much more influence on the PH than Tmax. IA treatment was more likely to be performed in patients with severe neurological deficits and perfusion delay, and PH might reflect increasing stroke severity and more aggressive treatment (22). When all cases were treated with endovascular thrombolysis, the influence of aggressive treatment disappeared, and this may explain the discrepancy in findings between Yassi's study and our study. Secondly, the correlations between DT >2 s, >4 s, >6 s, >8 s, and >10 s were statistically high from 0.906 to 0.992; therefore, the results might also be much dependent on different study populations. Moreover, although the thresholds at more extreme reductions in CBF, CBV, Tmax, and regional very low CBV have been previously shown to be useful in MRI, they were to be of limited utility using CTP, mainly because of a lack of sensitivity to very low levels of contrast in the severely hypo-perfused region, which produces a relatively large region of undetectable CBF and CBV.

Reperfusion of the ischemic core is an important cofactor in the pathogenesis of hemorrhage (10, 28). The CBV <2 mL/100 g, and CBF < 40% could be equal to the ischemic core in previous studies(17, 29). Howvere, in this study, CBF < 40% and CBV <2 mL/100 g could not be able to predict PH statistically in ROC analysis. Poor collaterals and recanalization were also found to correlate with HT (20), but in this study, we were unable to conclude that the poor collateral circulation and therapeutic recanalization had more PH. This study found that DT >4 s may be a better predictor of PH after IA tPA thrombolysis therapy. Therefore DT >4 s reflect perhaps both collaterals and reperfusion, and indicate that there is still some blood reaching the tissue, independent of the source of these collaterals or reperfusion. Additionally, instead of having to consider collaterals, reperfusion, and severe ischemia, now a single parameter can be assessed: DT >4 s.

The ECASS III trial used the following definition for symptomatic HT: any blood in the brain or intracranially associated with a clinical deterioration 4 NIHSS points that was identified as the predominant cause of neurological deterioration (30). However, it is often difficult to differentiate the predominant cause of neurological deterioration in clinical practice (22). The different clinical outcomes after different subtypes of HT illustrates the difficulty in defining symptomatic HT precisely and clearly (11, 31). Also, patients with HI often showed no symptoms. Thus, in this study, we used the PH as the outcome of symptomatic HT.

Limitations include the modest numbers of patients in the cohort. Due to the retrospective nature, PH was measured in different time intervals. There was a significantly increased mortality rate of our cohort, especially in those patients with PH2. It might be related to the prolonged treatment window time and more severe state of illness as the mean NIHSS was greater than 15. Its small sample size hampers proper multivariate analysis. Second, the patient cohort was collected from three different hospitals retrospectively, so inconsistency on imaging parameters may exist, although the CT scanners were all 64-slice scanners and used the same cine mode, and the iodinated contrast material was the same brand with similar injection methods. Also, the whole brain was not covered in the CTP studies, as 64-slice CT scanners were used in this study (32). And dual-energy CT was not used in this study. Third, quantitative analysis of other values of perfusion maps (CBF, MTT, CBV) with a range of thresholds was not included in our study. We chose to focus on DT on the basis of previously published data indicating its value as a predictor of PH (15).

In conclusion, the results of this study indicate that the moderate perfusion delay rather than severe delay was independently associated with PH after endo-vascular thrombolysis. Although the ischemic core on CTP is useful in the pretreatment prediction of HT, the moderate hypo-perfusion on DT is more strongly associated and may allow better prediction of PH after endovascular thrombolysis. Perfusion imaging may be significant not only for the fate of cerebral tissues, but also for the prevention of PH. Further studies are needed for a better understanding of the pathogenesis of PH.

## Ethics statement

All procedures performed in the studies involving human participants were in accordance with the ethical standards of the institutional review boards of PLA Army General Hospital and with the 1964 Helsinki Declaration and its later amendments or comparable ethical standards.

## Author's note

The datasets generated during and/or analyzed during the current study are available from the corresponding author on reasonable request.

## Author contributions

BW: study concept and design, analysis and interpretation of data, statistical analysis. NL: study concept and design, acquisition of data. MW, MP, HC, LL, YL, and ZS: study concept and design, critical revision of manuscript. SZ, GH, YZ, and JH: acquisition of data, analysis and interpretation of data. XW and GZ: study concept and design, critical revision of manuscript for intellectual content.

### Conflict of interest statement

ZS was employed by company GE Healthcare, Beijing. The remaining authors declare that the research was conducted in the absence of any commercial or financial relationships that could be construed as a potential conflict of interest.
