# Supplementary material for: Optimal Delay Time of CT Perfusion for Predicting Cerebral Parenchymal Hematoma After Intra-Arterial tPA Treatment
Source: Front Neurol. 2018 Aug 21;9:680. doi: 10.3389/fneur.2018.00680 (PMC6110878; doi:10.3389/fneur.2018.00680)
Supplement: Supplementary file 1 [file Table_1.DOCX]

Supplements:

Supplementary Table 1. Demographic Parameters and Other Relevant Information (n=94)

| **Clinical and imaging characteristic** | **Type of HT** | | | | | ***P* value** |
| --- | --- | --- | --- | --- | --- | --- |
|  | **No HT** | **HI-1** | **HI-2** | **PH-1** | **PH-2** |  |
|  | **(n=46)** | **(n=9)** | **(n=16)** | **(n=14)** | **(n=9)** |  |
| **Male, %** | **27(58.7)** | **2(22.2)** | **11(68.8)** | **11(78.6)** | **3(33.3)** | **0.0383 *** |
| **Age, ys** | **70.0 (52.0 -77.0 )** | **74.0(66.0-77.0)** | **65.0(53.0-71.5)** | **70.0(55.0-73.0)** | **62.0(56.0-72.0)** | **0.4747** |
| **NIHSS score at admission** | **15.5 (10.0 -19.0 )** | **16.0(12.0-18.0)** | **15.5(11.0-19.5)** | **16.5(13.0-19.0)** | **17.0(13.0-19.0)** | **0.6686** |
| **Hypertension,%** | **30(65.2)** | **6(66.7)** | **8(50.0)** | **10(71.4)** | **4(44.4)** | **0.5651** |
| **Diabetes,%** | **6(13.0)** | **1(11.1)** | **3(18.8)** | **4(28.6)** | **0(0.0)** | **0.3986** |
| **Hyperlipidmia,%** | **17(37.0)** | **1(11.1)** | **6(37.5)** | **3(21.4)** | **0(0.0)** | **0.0382 *** |
| **Atrial fibrillation,%** | **9(20.0)** | **5(55.6)** | **5(31.3)** | **3(21.4)** | **0(0.0)** | **0.0416 *** |
| **CAD,%** | **11(25.6)** | **2(22.2)** | **2(12.5)** | **1(7.1)** | **2(22.2)** | **0.5117** |
| **Current statin administration,%** | **13(28.3)** | **1(11.1)** | **5(31.3)** | **1(7.1)** | **2(22.2)** | **0.3282** |
| **Time from onset to CT imaging, h** | **5.3 (3.5 -7.8 )** | **5.0(4.0-8.0)** | **5.5(3.1-7.3)** | **6.3(3.0-9.5)** | **5.5(4.8-11.5)** | **0.6997** |
| **ASPECTS score on NCT** | **9.0 (8.0 -10.0 )** | **7.0(6.0-8.0)** | **7.0(6.0-9.5)** | **7.5(7.0-9.0)** | **7.0(6.0-8.0)** | **0.0438*** |
| **HMCAS on NCT, %** | **21(50.0)** | **6(75.0)** | **8(61.5)** | **10(71.4)** | **7(77.8)** | **0.3461** |
| **Collateral Scores** | **3.0 (2.0 -3.0 )** | **2.0(2.0-2.0)** | **1.0(1.0-2.0)** | **2.5(2.0-3.0)** | **2.0(2.0-3.0)** | **0.0009*** |
| **DT>2, mL** | **55.0 (47.0 -70.7 )** | **56.8(54.3-65.8)** | **55.5(31.8-71.6)** | **57.9(44.4-68.1)** | **60.9(55.2-70.2)** | **0.8553** |
| **DT>4, mL** | **27.6 (12.7 -38.8 )** | **26.0(23.4-37.4)** | **32.2(16.3-40.4)** | **35.1(19.8-54.3)** | **39.2(31.3-50.3)** | **0.1923** |
| **DT>6, mL** | **10.7 (1.6 -21.3 )** | **12.8(6.8-18.5)** | **14.3(2.4-22.2)** | **16.1(8.1-34.2)** | **21.7(14.1-29.2)** | **0.2819** |
| **DT>8, mL** | **2.9 (0.3-10.1)** | **5.8(2.0-9.0)** | **6.4(0.9-10.4)** | **4.2(2.1-21.0)** | **7.1(5.4-15.8)** | **0.3635** |
| **DT>10, mL** | **0.7 (0.0-4.0)** | **1.9(1.8-5.5)** | **1.5(0.1-4.6)** | **1.5(0.0-11.4)** | **3.3(2.1-10.1)** | **0.3672** |
| **rCBF<40%, mL** | **13.3(2.6-19.8)** | **18.0(10.5-25.7)** | **19.2(8.4-27.6)** | **18.6(8.7-30.5)** | **16.9(5.6-30.5)** | **0.2957** |
| **CBV<2, mL** | **11.7 (0.7 -24.7 )** | **21.7(4.0-33.6)** | **29.6(17.0-36.1)** | **24.9(16.5-32.8)** | **7.9(3.6-21.9)** | **0.0427 *** |
| **Site of occlusion** |  |  |  |  |  |  |
| **ICA, %** | **3(6.5)** | **0(0.0)** | **2(12.5)** | **2(14.3)** | **1(11.1)** | **0.2268** |
| **M1, %** | **35(76.1)** | **9(100.0)** | **9(56.3)** | **8(57.1)** | **5(55.6)** |  |
| **ICA & M1, %** | **8(17.4)** | **0(0.0)** | **5(31.3)** | **4(28. 6)** | **3(33.3)** |  |
| **Successful recanalization** | **38(82.6)** | **7(77.8)** | **12(75.0)** | **12(85.7)** | **7(77.8)** | **0.9438** |
| **Died cases (rate)** | **12(26%)** | **3(33%)** | **3(19%)** | **2(14%)** | **4(44%)** | **-** |

ASPECTS indicates Alberta Stroke Program Early CT Score; CAD, coronary artery disease; CT, computed tomography; HMCAS, hyperdense middle cerebral artery sign; NCT, noncontrast-CT; NIHSS, National Institutes of Health Stroke Scale; DT, delay time; HT, hemorrhagic transformation; HI, hemorrhagic infarction; PH, parenchymal hematoma; rCBF, relative cerebral bold flow, CBV, cerebral blood volume; ICA, internal carotid artery; M1, middle cerebral artery.

*** *P*<0.05**

Supplementary Table 2. Receiver Operating Characteristic Analysis

|  |  | Any HT | | | | |
| --- | --- | --- | --- | --- | --- | --- |
|  |  | AUC | 95% CI | | *P* Value | |
| DT>2s |  | 0.529 | 0.412 | 0.647 | | 0.623 |
| DT>4s |  | 0.606 | 0.492 | 0.721 | | 0.075 |
| DT>6s |  | 0.609 | 0.495 | 0.723 | | 0.068 |
| DT>8s |  | 0.601 | 0.486 | 0.716 | | 0.092 |
| DT>10s |  | 0.587 | 0.472 | 0.703 | | 0.145 |
| rCBF<40% |  | 0.632 | 0.520 | 0.745 | | 0.027* |
| CBV<2 |  | 0.650 | 0.539 | 0.761 | | 0.012* |

DT indicates delay time; AUC, area under the curve; CI, confidence interval; rCBF, relative cerebral bold flow, CBV, cerebral blood volume;

* P<0.05

| Variable | Univariate Model | |
| --- | --- | --- |
|  | Odds Ratio (CI) | *P* Value |
| NIHSS | 1.07(0.98-1.18) | 0.152 |
| Age | 1.00(0.96-1.03) | 0.795 |
| DT > 4 s, mL**^a^** | 1.04(1.01-1.06) | 0.013* |
| Poor collaterals and recanalization | 1.03(0.60-1.75) | 0.928 |
| Time from onset to CT imaging | 0.996(0.951-1.042) | 0.853 |
| Hyperlipidemia | 0.294 (0.079-1.088) | 0.067 |
| HMCAS | 2.267(0.789-6.510) | 0.128 |

Supplementary Table 3. Univariate Logistic Regression Analysis for PH
